# *In Vivo* Caprine Model for Osteomyelitis and Evaluation of Biofilm-Resistant Intramedullary Nails

**DOI:** 10.1155/2013/674378

**Published:** 2013-06-11

**Authors:** Nhiem Tran, Phong A. Tran, John D. Jarrell, Julie B. Engiles, Nathan P. Thomas, Matthew D. Young, Roman A. Hayda, Christopher T. Born

**Affiliations:** ^1^Department of Orthopaedic Surgery, Alpert Medical School, Brown University, Suite 200, 2 Dudley Street, Providence, RI 02905, USA; ^2^Weiss Center for Orthopaedic Trauma Research, Rhode Island Hospital, Providence, RI 02903, USA; ^3^BioIntraface Inc., North Kingstown, RI 02852, USA; ^4^Department of Pathobiology, New Bolton Center, School of Veterinary Medicine, University of Pennsylvania, Kennett Square, PA 19348, USA

## Abstract

Bone infection remains a formidable challenge to the medical field. The goal of the current study is to evaluate antibacterial coatings *in vitro* and to develop a large animal model to assess coated bone implants. A novel coating consisting of titanium oxide and siloxane polymer doped with silver was created by metal-organic methods. The coating was tested *in vitro* using rapid screening techniques to determine compositions which inhibited *Staphylococcus aureus* growth, while not affecting osteoblast viability. The coating was then applied to intramedullary nails and evaluated *in vivo* in a caprine model. In this pilot study, a fracture was created in the tibia of the goat, and *Staphylococcus aureus* was inoculated directly into the bone canal. The fractures were fixed by either coated (treated) or non-coated intramedullary nails (control) for 5 weeks. Clinical observations as well as microbiology, mechanical, radiology, and histology testing were used to compare the animals. The treated goat was able to walk using all four limbs after 5 weeks, while the control was unwilling to bear weight on the fixed leg. These results suggest the antimicrobial potential of the hybrid coating and the feasibility of the goat model for antimicrobial coated intramedullary implant evaluation.

## 1. Introduction

Stabilization of traumatic injuries by intramedullary nailing is a very commonly used technique for the treatment of long bone fractures. However, fracture repair and healing may be complicated by the presence of open wounds, severe soft-tissue injury, and environmental contamination. These increase the potential for deep wound infection/osteomyelitis and the development of delayed union/nonunion [1–13].

While titanium and stainless steel implants have been the standard for fracture fixation, neither material provides active resistance to bacterial infection nor prevents biofilm formation [14].

Several types of surface treatments to reduce bacterial growth and infection have been investigated, including the introduction of nanosurface topography, manipulation of surface chemistry, and the permanent attachment of antibiotic drugs to implants to inhibit bacterial attachment, growth and resulting biofilm formation. Nanosurface modifications are well developed and have been demonstrated to facilitate healing and reduce the growth of bacteria, but not at the log scale level [15]. Antoci et al. developed methods for permanently attaching antibiotic drugs to medical devices with good *in vitro* results against bacteria [16], but raise the concern of preferentially selecting and encouraging development of drug-resistant bacterial infections. 

Silver has been used as an antibacterial material since ancient time. Currently, it is used for burn wound treatment, dental work, catheters, and bacterial infection control, in the form of metallic silver, silver nitrate, and silver sulfadiazine. Due to the recent increased number of antibiotic-resistant bacteria, silver has been studied extensively as an alternative for antibiotics. Bactericidal effects of silver depend greatly on concentration [17–19], particle size [20], and shape [21] in the case of silver particles. Exposure to silver causes changes in bacteria cell membrane morphology and damage to intracellular proteins such as DNA, which affect cell metabolism, cell division, and can result in cell death [20].

Recently, Jarrell et al. used titanium siloxane hybrid to control the release of vanadium for soft-tissue integration [22]. In this study, we report dose-dependent responses of bone forming cells (osteoblast) and bacteria (*S. aureus*) to hybrid coatings doped with silver. The current study also aims to create a large animal model for osteomyelitis and the evaluation of antimicrobial coated intramedullary nails utilizing a goat tibia osteotomy model. A goat model was chosen because of larger intramedullary canals which permits testing of human size fracture fixation devices such as intramedullary nails.

## 2. Materials and Methods

### 2.1. *In Vitro* Experiments

#### 2.1.1. Materials

Titanium oxide and polymer hybrid surface treatment solutions (BioIntraface, North Kingstown, RI, USA) are a patented technology synthesized using metal-organic chemistry and doped with metal-organic precursors to form silver oxide dispersions within the matrix [23].

#### 2.1.2. Preparation of Coatings

Stock precursor solutions for titanium oxide, siloxane, and silver were allowed to age 15 min at room temperature and briefly shaken before use. These stock solutions were added together in a separate glass container using a pipette to make hybrid stock solutions of specific compositions and briefly shaken before use. Stock solutions were mixed for titanium-siloxane hybrids and were subsequently doped with increasing amount of silver neodecanoate. Tertiary coatings are reported as weight% of silver in the coating. A series of solutions with silver concentration ranging from 0 to 18.26 wt% was prepared. A solution consisting of 100% silver neodecanoate was used as positive control. The surface treatments were applied directly to the bottom of tissue-culture-treated polystyrene (PS) 96-well microplates (Corning, Lowell, MA, USA), air dried, and heat treated on a hotplate to 95°C for one hour.

#### 2.1.3. Primary Osteoblast Proliferation Assays

For human cell assays, primary osteoblasts were cultured in primary cell culture medium (ScienCell Research Laboratories, Carlsbad, CA, USA) until being nearly confluent. Cells were then collected and seeded on hybrid coated 96-well plates at a density of 5000 cells/well. Cells were then incubated in a standard culturing condition (37°C, 5% CO_2_).

Osteoblast proliferation and viability on surface treatments was measured using standard colorimetric WST-1 assay (Roche Applied Science, Indianapolis, IN, USA). For WST-1 assay, after 2 days of incubation, each well of the microplate was filled with 10 *μ*L of WST-1 reagent. The microplate was incubated at 37°C, 5% CO_2_ for an additional 4 hours. After the indicated time, the optical density was measured at 450 nm using a microplate reader (SpectraMax Plus 384 Spectrophotometer, Molecular Devices, Sunnyvale, CA, USA). Measured absorbance was subtracted to absorbance of the same well without cells and normalized to optical density of polystyrene control.

#### 2.1.4. Bacteria Adhesion and Grow Assays

For the bacterial adhesion and growth assays, *S. aureus *(ATCC 25923, Manassas, VA, USA) was propagated in 5 mL Luria Broth (LB, Sigma, St. Louis, MO, USA) overnight and aliquot into 2 mL vials with 10% glycerol in fresh LB and kept at −80°C. Two days before experiments, bacteria were streaked from a frozen vial onto a LB agar plate and incubated at 37°C for 1 day. A single colony of *S. aureus* was selected from the agar plate and propagated in LB overnight to reach an optical density of 1.11 at 578 nm which corresponds to 2 × 10^11^ CFUs/mL. Bacteria were diluted 1000 times using fresh LB and seeded onto the hybrid surface treatments on microplate wells and incubated for 20 minutes. The wells were then rinsed with 200 *μ*L PBS to remove nonadhered bacteria. 100 *μ*L fresh LB was added to each well and incubated at 37°C. After 1-day incubation, optical density of each well at 578 nm was measured using a microplate reader to determine bacteria growth.

For each experiment, at least six replicates were used for each coating concentration. Statistical analysis was done by Student's *t*-test and data are expressed as mean ± standard deviation (SD).

### 2.2. *In Vivo* Goat Model

#### 2.2.1. Intramedullary Nail Coating

On preparation for a large animal study, the surface treatment was dip-coated onto 316LVM stainless steel intramedullary nails, 8 mm × 200 mm (Surgical Implant Generation Network (SIGN)). The nails were withdrawn from the solution and air-dried at room temperature before being packaged and autoclaved for implantation. The interlocking screws were also coated using the same technology. One unused coated screw and one coated screw retrieved following the actual experiment were analyzed using X-ray photoelectron spectrometer (XPS) to confirm the presence of silver (Ag) on the surface before and after the experiments.

#### 2.2.2. Animal Selection

The study was approved by the Brown University Institutional Animal Care and Use Committee (IACUC). Two mature female goats (1 control and 1 treatment), with a weight range of approximately 40–50 kg, were used for this study.

#### 2.2.3. Surgical Procedure

Radiographs of each operative tibia were obtained immediately prior to surgery. After surgical draping, the tibia was exposed through a 2 cm incision over the medial aspect of the stifle and tibia. No systemic antibiotics were given preoperatively or postoperatively. A simulated open tibial fracture was created in the mid-diaphysis with a sagittal saw cooled by saline irrigation.

Standardized suspensions of *S. aureus *(ATCC 25923) were diluted in phosphate buffer saline (PBS) to a final inoculum concentration of 2 × 10^4^ colony forming units per milliliter (CFU/mL). Bacterial colony counts were confirmed by culture using a spread-plate technique, in duplicate, on tryptic soy agar. One milliliter of bacterial inoculum was injected into the medullary canal at the osteotomy site.

The osteotomy was then stabilized by internal fixation using the stainless steel alloy intramedullary nail with proximal and distal interlocking screws. The 8 mm nail was inserted after the medullary canal had been reamed to cortical bone with flexible intramedullary reamers. Reaming was carried out in 0.5 mm increments to 9.5 mm. Final insertion of interlocking screws was then performed using the jig for the SIGN nail system. The infrapatellar incision was irrigated with normal saline solution and closed in layers with 3–0 Vicryl sutures (polyglycolic acid; Ethicon).

#### 2.2.4. Postoperative Care

Postoperative pain management included application of a fentanyl patch to the neck of each goat. Examination of the goats by the veterinary staff was performed prior to the removal of the patches.

The incision sites from the osteotomy were evaluated weekly for inflammation. At each inspection, the condition of the wound sites was photographed. Two 10 mL vials of blood (20 mL total) were drawn from peripheral vessels under sedation and tested for bacteria, complete blood count and elemental analysis pre- and postoperatively, at weeks 1, 2, 4, and prior to sacrifice. The silver and titanium levels were measured in the whole blood by Inductively Coupled Plasma (ICP) spectrometry at the time of sacrifice.

#### 2.2.5. Samples Collection

At five weeks, the animals were sacrificed by a lethal overdose of barbiturate (Beuthanasia-D). Seven organs, including liver, heart, spleen, brain, kidney, gallbladder, and small intestine, were collected and preserved in 10% formalin solutions. The organ samples were then shipped to Brooks Rand Labs (Seattle, WA, USA) for silver and titanium levels analysis using Inductively Coupled Plasma mass spectrometry (ICP-MS) technique.

The hind limbs were harvested and preserved in 70% ethanol solutions for further mechanical tests and histology.

#### 2.2.6. Radiographic Evaluation

Craniocaudal (anteroposterior) and lateromedial radiographs of the tibia were taken preoperatively, immediately post-operative, and at postoperatively intervals at weeks 1, 2, 4, and 5 using general anesthesia. The radiographs were coded and evaluated in a blinded fashion by an experienced orthopaedic radiologist not affiliated with the study, who graded the callous formation, the stability of the bone, alignment, and appearance of fixation.

#### 2.2.7. Mechanical Tests

The implanted nail was removed from each harvested tibia utilizing an MTS 810 Servo hydraulic Load Frame augmented with custom fixtures. Load and displacement data were acquired using MTS flex test software (Minneapolis, MN, USA) at 200 Hz. Each specimen was pulled at a displacement rate of 4 mm/sec. The data were reported as ultimate load, displacement at ultimate load, and stiffness.

#### 2.2.8. Microfocal Computed Tomography (Micro-CT) Technique

After the IM nails were explanted, the tibias were cut down to 70 mm in length, and cross-sectional images were obtained using a computed tomography (CT) scanner (*μ*CT 40, Scanco Medical, Brüttisellen, Switzerland). During scanning, the bones were oriented in the scanner such that their sagittal, frontal, and transverse planes were aligned with the scanner's built-in coordinate system. Images were acquired using machine settings of 70 kV and 114 *μ*A. The resolution of the scanned images was 36 *μ*m (isometric voxel size). Three-dimensional images were reconstructed using ImageJ software (NIH).

#### 2.2.9. Histology

For histology, longitudinal sections of the tibia were taken from the fracture site, preserved in 70% ethanol, and prepared by methyl methacrylate processing and embedding and hard tissue sectioning. Sections were stained with hematoxylin and eosin (H&E), Toluidine Blue, and Gram stain. Samples were evaluated by a board-certified veterinary pathologist, who was blinded to the treatment and control animals.

A semiquantitative grading scheme was implemented to evaluate the extent of fracture healing, amount of bone lysis and bone necrosis, degree of inflammation, and presence of bacteria for both cortices. Individual scores obtained for each cortex were then added and divided by two to obtain an average score incorporating scores given for both cortices. Components of the bone assessed for fracture healing comprised the periosteal callus, osteotomy site, and cortical bone adjacent to the osteotomy site. The grading scheme is presented in Table 1, with the highest potential numerical score for each cortex were 65.

#### 2.2.10. Confocal Microscopy

Biofilm formation on the nail was assessed by confocal microscopy. After mechanical pullout testing, the nails were preserved in 70% ethanol solution. The tip of the nail was cut using a bolt cutter and placed in PBS. The nail tip was subsequently rinsed three times with PBS to remove unattached tissues on nail surface and transferred into a new vial containing 5 mL of 4′,6-diamidino-2-phenylindole (DAPI) 1 mg/mL solution (Invitrogen, Carlsbad, CA, USA) and FITC-conjugated *S. aureus* antibody solution (Thermo Scientific). After containment for 15 minutes at room temperature without exposure to light, the sample was rinsed three times with PBS to remove excess DAPI and antibody and imaged using a Confocal Laser Scanning Microscope (Zeiss LSM 510). Ten random 450 *μ*m × 450 *μ*m images on the nail surface were obtained. Due to the curvature of the nail, confocal microscopy was preferable to conventional fluorescent microscopy. The obtained fluorescent images were analyzed using ImageJ software for FITC and DAPI surface coverage.

## 3. Results

### 3.1. Osteoblast and *S. aureus* Response to Silver-Doped Ti/Siloxane Coating *In Vitro*

**Figure 1:**
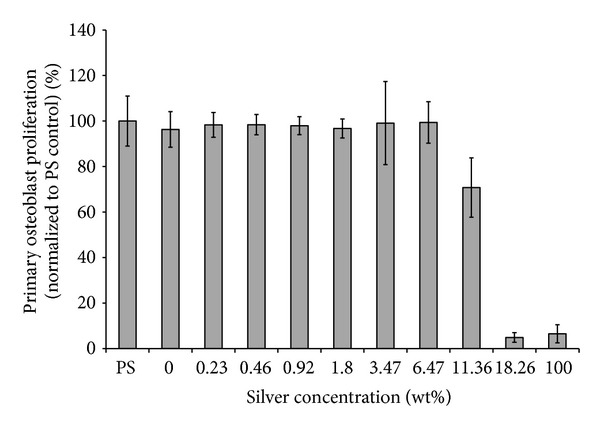
Osteoblast proliferation on the coating as a function of silver concentration by weight percent. The results are normalized to the noncoated polystyrene (PS) controls. Data = Mean ± SD.

Osteoblast viability and proliferation experiments were performed after two days of incubation with several concentrations of silver oxide in the coating. The results showed no reduction in cell proliferation for up to 11.36% silver doping of the coating (Figure 1). Cell density was lower on the substrates with 18.26% and 100% silver.

**Figure 2:**
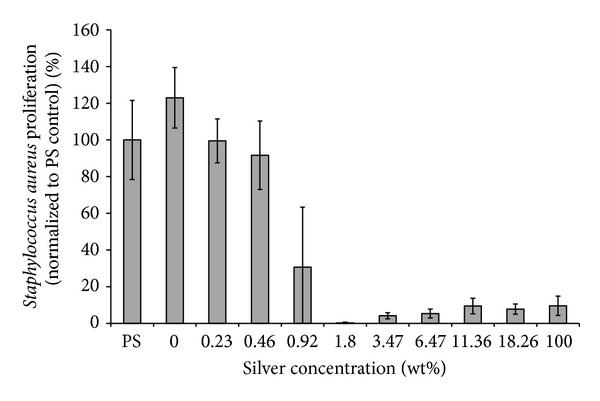
*Staphylococcus aureus *viability/proliferation on hybrid surface treatments after one day, as a function of sliver concentration by weight percent. The results are normalized to the noncoated polystyrene (PS) controls. Data = Mean ± SD.

Bacteria adhesion data showed that *S. aureus* growth was completely eliminated in the presence of 1.8% or more of silver in the coatings (Figure 2). A slightly increased optical density on samples with more than 1.8% silver was due to the elution of silver into the solution. The same plate with media but no bacteria was used to confirm this trend (data not shown). These results confirmed the antibacterial properties of the silver doped hybrid coatings. Moreover, there is a concentration window between 1.8% and 11.36% of silver in which the coatings do not affect osteoblast growth while still exhibiting bactericidal effects.

### 3.2. *In Vivo* Goat Model

#### 3.2.1. Nail and Screw Coating and Characterization

**Figure 3:**
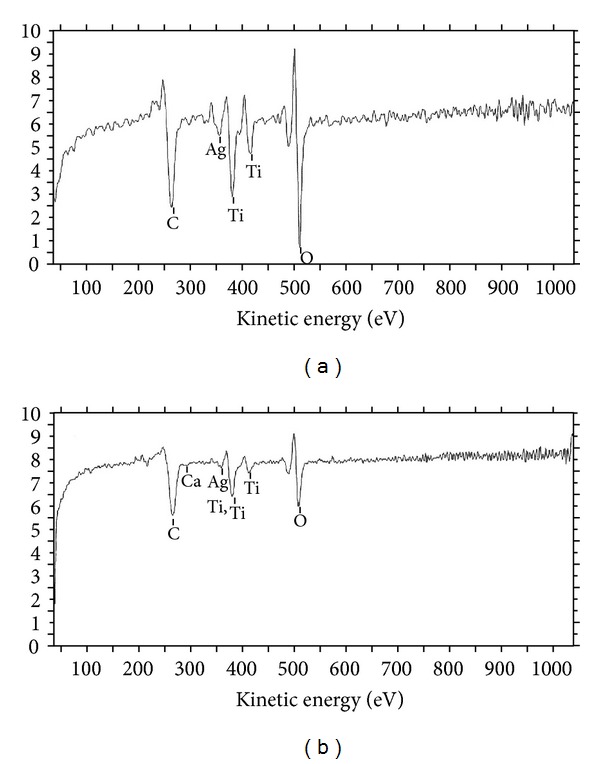
XPS analysis of coated interlocking screw. Silver was present on both unused (a) and used (b) screws. The analysis also confirmed the existence of Ti on the coated surface.

The XPS elemental analysis confirmed the presence of Ti and Ag on the coated sample (Figure 3). After explantation, the intensity of Ag peak on the surface decreased. The results indicated that silver was released from the coating. However, not all silver was released after 5 weeks of implantation.

#### 3.2.2. Clinical Observations

The fractures on treated and control goats were repaired successfully using IM nails without further complications. The fentanyl patch, which was used as pain reliever immediately after the operation, was removed from the treated goat two days after surgery; the patch on the control goat was removed one week after surgery.

After 5 weeks of implantation, the treated goat lost 7% of its initial body weight but was able to ambulate on the tested limb. In contrast, the control goat lost 8.4% of its weight and hesitated to bear weight on the tested limb, holding the limb in a flexed position high when ambulating. Subjective assessments regarding mentation, appetite, and general activity were more positive for the treated goat than the control goat.

#### 3.2.3. Microbiology Evaluation

Bacterial cultures from the proximal and distal screws collected after explantation were positive for *S. aureus* on the screws taken from both control and treated goat.

Throughout the study, blood samples were collected from both animals for aerobic culture. At all time points, 0, 1, 2, 4, and 5 weeks post-op, blood culture showed no aerobic growth in either goat (Table 2).

#### 3.2.4. Complete Blood Count

Complete blood counts of the control and treated goats are presented in Table 3. The white blood cell count (WBC) of the control goat did not change much after surgery. The WBC of the treated goat varied more but still in the normal range for goat (4–13 thousand/*μ*L). The neutrophil percentage of the control goat increased 151% 1 week after surgery. This neutrophil level was in high range until it eventually dropped in week 5. In contrast, the neutrophil level of the treated goat did not change much after surgery. The neutrophil level of treated goat also saw a decrease at week 5. The lymphocyte percentage followed the opposite trends compared to the neutrophil percentage in both animals. The normal ranges for neutrophil and lymphocyte in goats are 30–48% and 50–70%, respectively.

#### 3.2.5. Radiographs

**Figure 4:**
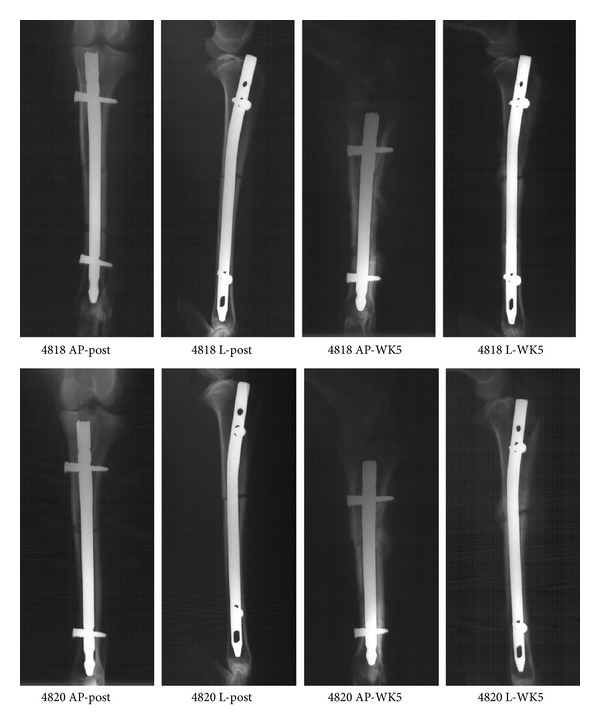
X-ray radiographs of the control (4818) and treated (4820) goat tibias immediately after surgery and 5 weeks after surgery. At each time point, craniocaudal (anteroposterior) and lateromedial views were taken. All images are at the same scale.

**Figure 5:**
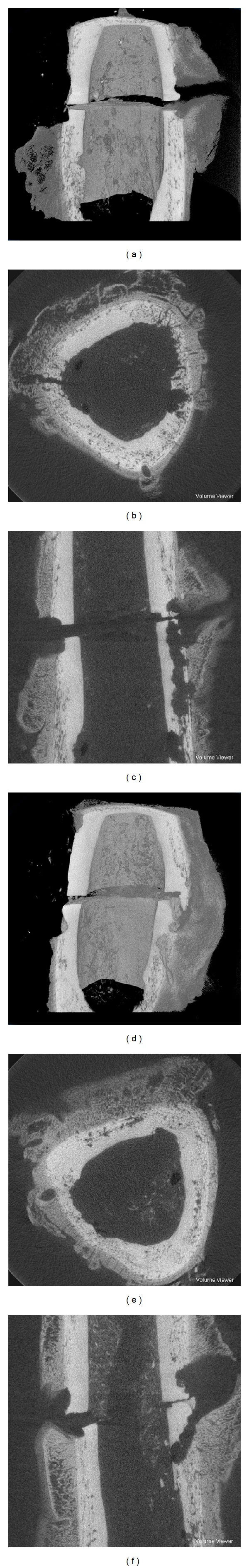
Micro-CT images of the tibia after the nails were removed. Images were taken at osteotomy sites and reconstructed using ImageJ. 3D reconstruction, top view and side view images for 4818 control goats ((a), (b), and (c)) and 4820 treated goats ((d), (e), and (f)) are presented.

X-ray and micro-CT images of goats tibias are shown in Figures 4 and 5, respectively. X-ray images demonstrate goats tibias post-op and before sacrifice. For each time point, anteroposterior (AP) and lateral (L) views were provided. According to these images, both goats were successfully stabilized using IM nails. There were no signs of implant loosening after 5 weeks. Periosteal reactions and infection were observed in both animals. The micro-CT images showed clear sign of bone lysis and necrosis due to bacterial infection.

Using the criteria shown in Table 4, the radiographs of goats tibias at 5 weeks were assessed. There was little radiographic difference between the control and the treated goats. The osteotomies were not healed. Periosteal reaction and osteolysis were observed, but without signs of sequestrum and implant loosening in either goat.

#### 3.2.6. Mechanical Testing

The mechanical testing results are presented in Table 5. The ultimate load for the treated goat was much higher than the value of the control goat. Testing also showed that the coated nail was more difficult to remove from its respective tibia than the noncoated nail, possibly indicating more bacterial infection in control goat or higher osseointegration in the treated goat.

#### 3.2.7. Histology

**Figure 6:**
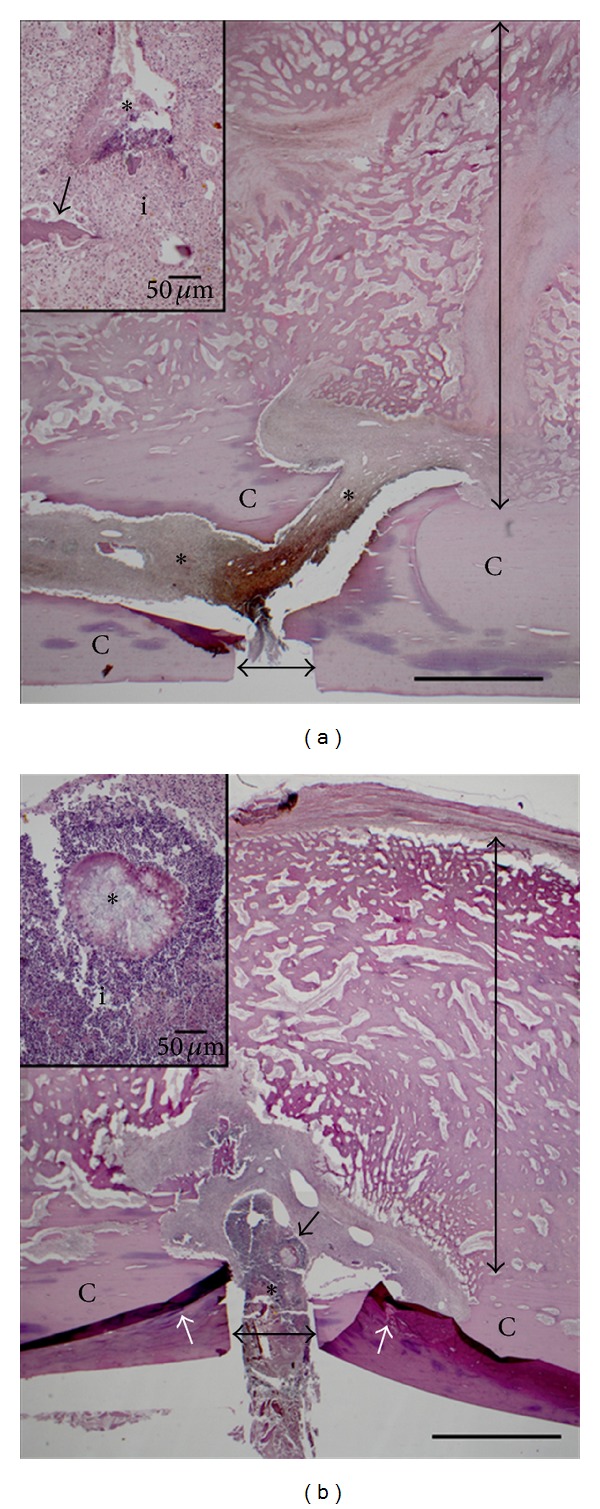
Composite photomicrographs of H&E stained longitudinal sections from the tibias cortex of control goat (4818) (a) and treated goat (4820) (b). 2X magnification photomicrographs (scale bars = 1 mm) show large periosteal callus (a,b—vertical double-headed arrows) composed of woven bone and fibrous connective tissue surrounding the osteotomy sites, which are not bridged by callus (a,b—horizontal double-headed arrows). Lysis of adjacent cortical bone (a,b—C) is evident, and inflammation (a,b—asterisks) concentrated at the osteotomy site dissects through the cortex of 4818. Folds in the cortex of 4820 (b, white arrows) are artifacts of processing. 20X magnification photomicrographs (insets, scale bars = 50 *μ*m) show necrotic debris (a, inset—asterisk) surrounded by inflammation (a, inset—i) and microsequestra (a, inset—arrow). A large bacteria colony surrounded by radiating arrays of proteinaceous material (b, inset—asterisk), consistent with Splendore-Hoeppli formation is surrounded by inflammation (b, inset—i).

Histological scores are presented in Table 6, and representative photomicrographs of bone from each animal are presented in Figure 6. Multifocal bacteria colonies were observed within both treated and untreated goats; however, average and individual scores for the tibial cortices of the treated goat (4820) were higher than the scores for the control goat (4818). Large areas of necrotic bone were observed far from the osteotomy site (>20 mm distance) in both cortices of the control goat compared to one cortex of the treated goat, and inflammation involving the adjacent cortical bone was more severe in the control than treated goat. Both animals had prominent, disorganized periosteal callus and incomplete bony union with a large component of fibrovascular connective tissue. Neither animal formed a bridging callus that filled the osteotomy gap. Bony sequestra were few (1–3) and small (<2 mm diameter) for both animals. Using the histology criteria in Table 5, the average score of the treated goat (4820) was 16.5 points, slightly higher than that of the control goat (4818), 10 points.

#### 3.2.8. Silver Level in Goat Organs and Blood

The silver levels in all organs harvested from the control goat (4818) were lower than the method detection limits of the measurements. Silver level in liver of the treated goat (4820) was detected to be 0.045 ppm, which was higher than the method detection limit (0.02 ppm) but still lower than the method reporting limit (0.099 ppm). No silver was detected from other organs of the treated goat. The results indicated that some silver was released from the coating into the goat body, but the silver level was very low. 

No silver or titanium was detected in any goat serum samples.

#### 3.2.9. Confocal Microscope Images

**Figure 7:**
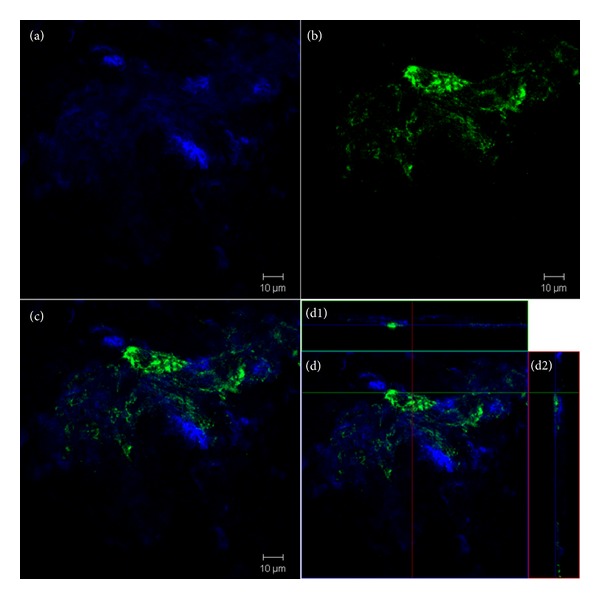
Representative confocal images of biofilm and tissue formed on an IM nail surface. Blue channel (a) and green channel (b) represent DAPI nucleus staining and FITC conjugated *S. aureus* antibodies. (c) and (d) are overlay images of both channels with 3D projection on *x* (d1) and *y* (d2) axes.

Fluorescent labeling was performed using DAPI nucleus staining and FITC-conjugated *S. aureus* antibodies. Representative images are presented in Figure 7, in which two fluorescent channels (blue: DAPI; green: FITC) were utilized. Biofilm was clearly differentiated from any native animal tissue that grew on the surface of the IM nail.

**Figure 8:**
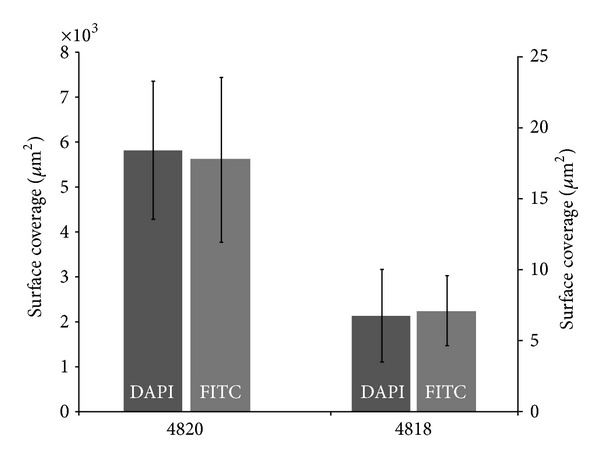
DAPI and FITC surface coverage on IM nails explanted from treated goat (4820) and control goat (4818). The surface coverage for DAPI (dark grey) is higher and is expressed using the left *y*-axis. The surface coverage for FITC (light grey) is showed using the right *y*-axis. Data = Mean ± SEM. Averages were taken from 10 random 450 *μ*m × 450 *μ*m images.

Surface coverage for DAPI and FITC was calculated using ImageJ software. Average coverage per a 450 *μ*m × 450 *μ*m area on IM nail surface is shown in Figure 8. Not many colonies of bacteria were found on surface of both control and treated nails. The treated surface had slightly more DAPI and FITC coverage. However, since the number of colonies was low and distributed nonhomogenously, the error of the measurement was high and the differences were not statistically significant.

## 4. Discussion

In this study, a nondrug antibacterial coating using silver-doped metal oxide/polymer hybrid was assessed. The materials were first coated onto a 96-well plate for *in vitro* assessment. As expected, there was a dose-dependent trend of antibacterial effect of the coating. With higher than 1.8% silver in the coating, *S. aureus* proliferation was reduced significantly compared to the control. It is also important to note that cell proliferation experiments were performed with the same materials using primary osteoblasts. The results showed that reduced cell proliferation was observed in coatings with 11.36% and higher amount of silver. Therefore, a coating with silver concentration in the window ranging from 1.8% to 11.36% will be effective for inhibiting *S. aureus* proliferation without reducing osteoblast growth *in vitro*.

In the current study, the technology was transferred from *in vitro* to *in vivo*. The antibacterial coating was tested using a goat osteotomy model. To our knowledge, this is the first study of its kind to look at antibacterial coated IM nails in a large animal model. Recently, Schaer et al. and Stewart et al. performed a similar, important study on antibiotic coated plates in a sheep model [24, 25]. However, we believe that developing an IM nail model is crucial because of the popularity of IM nails used in human trauma cases.

In this study, the simulated tibia fracture was induced and *S. aureus* was used to cause infection. After 5 weeks, both goats showed signs of infection as supported by lameness, reduced body weight, differential WBC counts, diagnostic imaging, and histopathologic findings. Aspirate from both the screw sites and the fracture sites showed microorganisms and were confirmed to be *S. aureus*. Therefore, the amount of bacteria used in this study (2 × 10^4^ cfu) was enough to generate infection in goat tibia. In fact, further study should consider using fewer bacteria and a better inoculate technique. Infection in soft tissue reduced the effectiveness of the coating and resulted in nonreliable quantitative data. 

The mechanical pullout testing experiments showed higher ultimate load to remove the treated nail compared to the control nail. These data could imply that the control goat had a more severe infection inside the medullary canal, which resulted in loosening around the nail. Alternatively, it might suggest that the treated goat had more bone in-growth onto the surface of the nail [26]. Combining radiographic, CT imaging, histology data, and the short duration of the study, a more severe infection in control goat is the more likely explanation. It is also possible that canal shape and curvature mismatch of the nail were responsible for different pullout strengths. Nevertheless, to obtain a more conclusive result, a larger population of samples would be desirable.

In our current study, silver was released in ionic form instead of nanoparticles. Many studies on silver nanoparticles have shown bactericidal effect on several different bacteria species [19, 21, 27, 28]. However, concerns have been raised regarding adverse effects when nanomaterials penetrate and interact with host cells [29–31]. The amount of silver released into the goats' blood and organs after 5 weeks in our study was negligible. Hence, a cytotoxic effect caused by the systemic release of silver is minimal or less likely.

A histology grading scheme was developed in this study. Using this detailed scoring system, both cortices of the control and the treated goat were evaluated. The treated goat received a higher score and exhibited less inflammation. However, a larger set of animals is needed to confirm any statistically significant difference of the treatment. 

In order to visualize the nail surface after removal from the goat tibia, confocal microscopy was utilized. Using this method, surface coverage of bacteria biofilm and tissue was quantified using image analysis. Both cells and bacteria were observed on control and treated nails. However, the differences in surface coverage were not statistically significant. More images can be obtained to improve the comparison. This method could also provide a 3-dimensional reconstruction of biofilm presented on nail surface, which can be further quantified using image analysis tool.

## 5. Conclusion

In the current study, a caprine model was developed to study osteomyelitis and infection resistant IM nails. Grading systems and analysis methods were established to evaluate a novel hybrid coating. The goat that received a coated IM nail was more healthy and able to walk better after 5 weeks of treatment. The treated goat also received better histology score. However, only two goats were used to create this model. A larger set of animals with adjusted inoculation amount and methodology will likely improve the statistical differences between the treatments. A method using confocal microscopy and fluorescent labeling was also presented to quantify biofilm formation on IM nail surfaces. With an increasing number of antibiotic resistant bacteria, alternative coatings (such as silver) for orthopaedic medical devices are needed. We hope that the model developed in this study will be useful for evaluating such coatings in a preclinical setting.

## Figures and Tables

**Table 1:** Histology scoring criteria.

Periosteal callus	
Subjective size and quality	Minimal/mild = 3; prominent, well organized = 2; prominent disorganized = 1; incomplete union = 0
Woven-lamellar bone percent	0–25% = 3; 25–50% = 2; 50–75% = 1; 75–100% = 0
Cartilage tissue percent	0–25% = 3; 25–50% = 2; 50–75% or incomplete union = 1; 75–100% or fibrous nonunion = 0
Fibrovascular tissue percent	0–25% = 3; 25–50% = 2; 50–75% = 1; 75–100% or fibrous nonunion = 0
Inflammation percent	0–25% = 3; 25–50% = 2; 50–75% = 1; 75–100% = 0
Osteotomy site	
Bridging bony callus percent	75–100% = 4; 50–75% = 3; 25–50% = 2; 10–25% = 1; 0–10 = 0
Woven-lamellar bone percent	0–25% = 3; 25–50% = 2; 50–75% = 1; 75–100% = 0
Mineralized bone percent	75–100% = 3; 50–75% = 2; 25–50% = 1; 0–25% = 0
Fibrovascular tissue percent	0–25% = 3; 25–50% = 2; 50–75% = 1; 75–100% or inflammatory exudate = 0
Inflammation percent	0–25% = 3; 25–50% = 2; 50–75% = 1; 75–100% = 0
Adjacent cortical bone	
Degree of osteolysis	None/minimal = 4; mild local expansion (remodeling) of osteonal canals = 3; <1 mm adjacent osteolysis = 2; 1–3 mm adjacent osteolysis = 1; >3 mm adjacent osteolysis = 0
Extent of bone necrosis	Empty lacunae and/or necrotic osteons: <2.5 mm = 4; 2.5–5 mm = 3; 6 mm–10 mm = 2; 11–20 mm = 1; >20 mm = 0
Extent of inflammation	None/minimal = 4; mild local ostonal canals inflammation = 3; inflammation >1 mm distance = 2; 1–3 mm distance = 1; >3 mm distance = 0
Presence of sequestra, for example, necrotic fragments of bone	None = 8; 1–3 small <2 mm = 4; >3 or single large >2 mm = 2; single to multiple large >5 mm = 0
Presence of bacteria	None = 8; rare small colonies = 4; multifocal colonies (<25 *µ*m) = 2; multifocal large colonies (>50 *µ*m) = 0

**Table 2:** Bacteria culture results from distal and proximal screw sites, fracture site, and blood.

Goats number	Distal screw site	Proximal screw site	Fracture site	Blood
4818 (control)	+++	+++	+++	−NEG
	*S. aureus *	*S. aureus* (2 biotypes)	*S. aureus* (2 biotypes)	
4820 (treated)	++	++++	+	−NEG
	*S. aureus* (2 biotypes)	*S. aureus *	*S. aureus *	

**Table 3:** Complete blood count showing white blood cell count (WBC), neutrophil percentage, and lymphocyte percentage at preoperation and weeks 1, 2, 4, and 5.

Time	4818 (control)			4820 (treated)		
	WBC (Thous./*µ*L)	Neutrophil (%)	Lymphocyte (%)	WBC (Thous./*µ*L)	Neutrophil (%)	Lymphocyte (%)
Pre-op	6.3	31	64	9.7	74	21
Week 1	6.2	78	22	6.2	61	38
Week 2	5.9	67	33	7.9	73	26
Week 4	6.4	79	21	6.3	76	24
Week 5	5.6	37	55	8.8	59	39

**Table 4:** Assessments from radiographs of treated and control goat tibias. The criteria here are bridging callus, periosteal reaction, osteolysis, sequestrum, implant loosening, and soft-tissue swelling.

Radiology criteria	4818 (control)	4820 (treated)
Bridging callus	<50% (1/4 cortices)	<50% (1/4 cortices)
Periosteal reaction	Present	Minimal
Osteolysis	Present (irregular osteolysis proximally)	Minimal
Sequestrum	Absent	Absent
Implant loosening	Absent	Absent
Soft-tissue swelling	Not evaluable	Not evaluable

**Table 5:** Mechanical testing results showed ultimate load and displacement to remove the intramedullary nail from goats, tibias after 5 weeks of implantation.

Mechanical testing criteria	4818 (control)	4820 (treated)
Ultimate load (N)	150.3	497.6
Displacement (mm)	8.7	2.7

**Table 6:** Histology scores for control goat (4818) and treated goat (4820) assessed for callus formation, osteotomy site, adjacent cortical bone, presence of sequestra, and bacterial persistence. Averages of the added scores from each cortex are given. Highest potential score is 65.

Histology criteria	Cortex 1 (4818)	Cortex 2 (4818)	Cortex 1 (4820)	Cortex 2 (4820)
Periosteal callus formation				
Subjective size and quality	1	1	1	0
Woven-lamellar bone	0	0	0	0
Cartilage (hyaline and fibrocartilage)	1	0	3	0
Fibrovascular tissue	2	0	3	0
Inflammation	3	1	3	2
Osteotomy site				
Bridging bony callus	0	0	0	0
Woven-lamellar bone	0	0	0	0
Mineralized bone	0	0	0	0
Fibrovascular tissue	0	0	0	0
Inflammation	2	0	0	0
Adjacent cortical bone				
Osteolysis	0	1	1	0
Osteon/osteocyte necrosis	0	0	4	1
Inflammation	0	0	1	4
Sequestra	4	4	4	4
Bacterial persistence	0	0	0	2

Total	13	7	20	13

Average	10		16.5	
